# Aligned-Layer Text Search in Clinical Notes

**Published:** 2017

**Authors:** Stephen Wu, Andrew Wen, Yanshan Wang, Sijia Liu, Hongfang Liu

**Affiliations:** a Department of Medical Informatics and Clinical Epidemiology, Oregon Health & Science University, Portland, OR, USA,; b Division of Biomedical Informatics, Mayo Clinic, Rochester, MN, USA

**Keywords:** Natural Language Processing, Information Storage and Retrieval, Electronic Health Records

## Abstract

Search techniques in clinical text need to make fine-grained semantic distinctions, since medical terms may be negated, about someone other than the patient, or at some time other than the present. While natural language processing (NLP) approaches address these fine-grained distinctions, a task like patient cohort identification from electronic health records (EHRs) simultaneously requires a much more coarse-grained combination of evidence from the text and structured data of each patient’s health records. We thus introduce aligned-layer language models, a novel approach to information retrieval (IR) that incorporates the output of other NLP systems. We show that this framework is able to represent standard IR queries, formulate previously impossible multi-layered queries, and customize the desired degree of linguistic granularity.

## Introduction

Search (i.e., information retrieval) techniques in clinical text face the challenge of coarse vs. fine granularity. In a setting such as electronic health records (EHRs), the goal is usually to find a broad characterization of a whole patient from that patient’s diverse assemblage of text and structured data. How to combine these pieces of evidence into a patient-level judgment is an area of active research. Moreover, in clinical text, dictionary matching for a symptom like “hypertension” must be augmented by fine-grained algorithms to ensure that the symptom is not negated (e.g., “no complaints of …”), about someone other than the patient (e.g., “family history of”), or a host of other possibilities. Textual variants of these fine-grained patterns abound as well, spawning natural language processing (NLP) methods to find concepts of interest from acceptable textual contexts.

Therefore, we aim to incorporate arbitrary NLP-derived features into information retrieval (IR) methods on clinical text, allowing for greater control of the granularity in search. Our main contribution is the *aligned-layer information retrieval* model, which we specify by defining the “layered” nature of text and the nature of phrases in this setting. This model is a straightforward extension of the language modeling approach to IR and its feature-centric successors.

We adopt the “layer” terminology from the language resources community in which, for example, treebanking and propbanking are different layers of annotations on the same text. The crucial contribution in our work is a model in which all text-derived “layers” are aligned by token, and can be utilized and scored simultaneously during search.

After describing our aligned layer approach, we report on a preliminary system implementation and evaluation of the model. For our evaluation, we focus on the problem of cohort identification within clinical notes from the EHR.

## Related Work

Preliminary explorations with concepts (from the Unified Medical Language System (UMLS)) in language modeling have met with moderate success [1–3] in the past. Language modeling in IR [4–6] builds on a rich tradition of probabilistic IR [7], and has a successful history of ranking documents based on well-motivated textual features. The most commonly-used textual features are from Metzler and Croft’s dependence model, modeling the probabilistic relationships between query terms (and a candidate document) as Markov Random Fields (MRFs). These term–term dependencies significantly outperformed the original bag-of-words language modeling approaches to IR from which they arose[4; 5]. Subsequently, query hypergraphs made it possible to model higher-order dependencies (e.g., multiword–multiword or multiword–term dependencies) [8]. While the representational power of these feature-based models allows for arbitrary *non-textual* features as well (e.g., named entities or dependencies, such as we introduce here), non-textual features have received minimal attention in the literature.

Recent work on joint text and concept search [2] and split-layer language models [1] extended the notion of mixing different document representations together [9; 10]. Arising out of the medical domain, these techniques all showed some benefit to considering multiple “layers” simultaneously. Our work here extends the discussion of language modeling layers, pushing the question of what semantic representations (or granularity thereof) are effective for IR.

Positional language models [11] attempt to model the intuition that closer textual proximity can correspond to greater association. This shares an important intuition with aligned-layer models: position is important in IR. However, our accounting of position is not to model the textual distance, but the content and relationships that are latent within text at the same locations. A number of existing studies [1; 2] require an NLP preprocessing step prior to indexing the collection, and utilize these text-derived features in search. However, these attempts fail to allow navigation among, and correspondences between, those structures.

The task of query formulation [12] is somewhat upstream task to our layered models; query formulation can include the introduction of term dependencies [13], weighting of terms [14], query expansion [15], and the parameterization of associated weights according to additional corpora [16; 17]. Our hope is that future query formulation techniques will make use of aligned-layer structures.

The TREC Medical Records track [18; 19] provided resources for significant innovation in medical IR, which used in this paper for evaluation: collections of clinical text, information needs, and judgments. Language modeling techniques have been successful in this clinical text setting [2], as well as in health-related web search exemplified by the CLEF eHealth challenges [20].

## Methods

**Figure 1 -:**
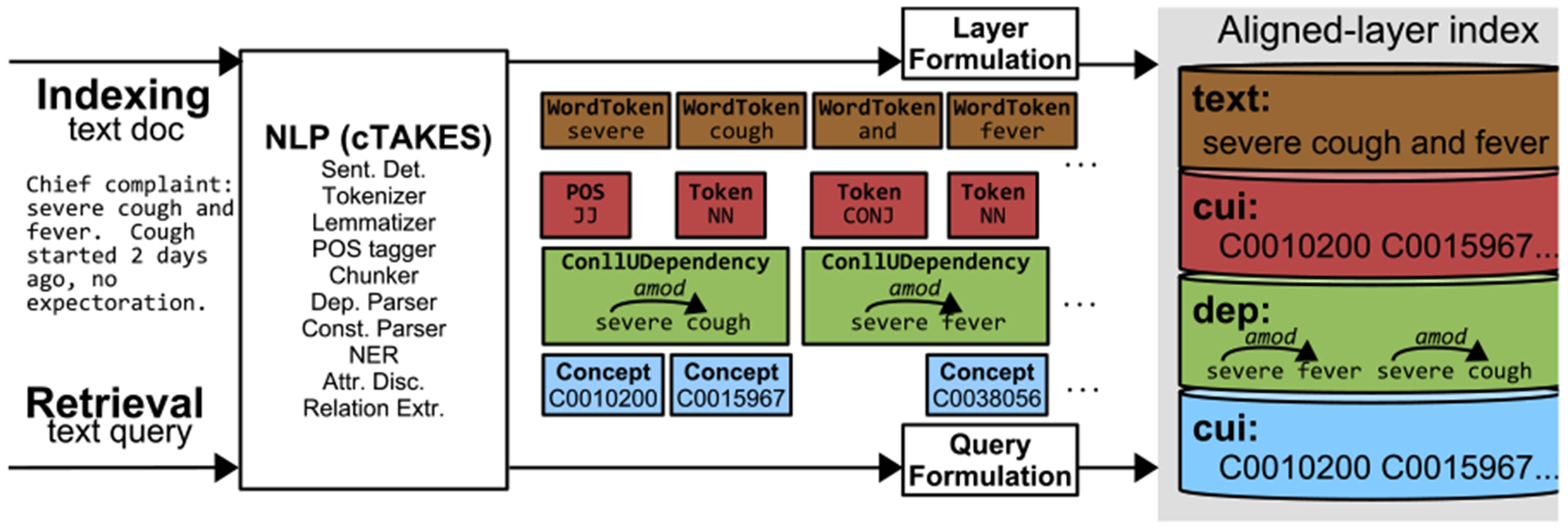
cTAKES processing is followed by the indexing of results into various layers (indexes)

We now turn our attention to the aligned-layer retrieval model and its implementation for our experiments. Let us assume that we run an NLP pipeline as a preprocess; e.g., the clinical Text Analysis and Knowledge Extraction System (cTAKES)^1^ [21]. Developed specifically for clinical text, this pipeline produces NLP artifacts as illustrated in the left and middle Figure 1.

### Indexing: Aligning text and NLP artifacts

We represent this NLP-preprocessed text as a finite sequence of multiple aligned layers. Specifically, a document or query is composed of multiple layers *L_0_*, *L_1_, L_2_*,… We reserve *L0* to be a *base layer* of the original text; namely, a sequence of tokens (optionally stopped and stemmed) by which all other layers are aligned.

**Figure 2 -:**
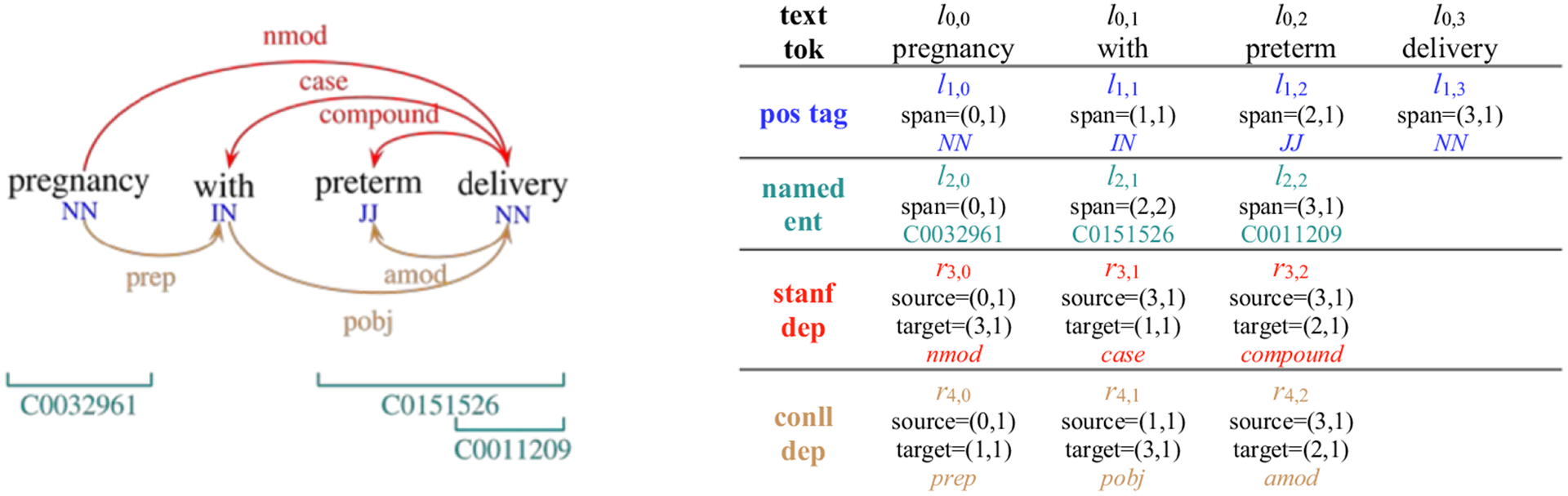
Aligned layers (tokens, part-of-speech tags, named entities, Stanford dependencies, and CoNLL-X dependencies) for a sample query “pregnancy with preterm delivery.” Span, source, and target indices are with respect to the L_0_ layer’s indices.

Figure 2 illustrates a query text (tokens) as a base layer, with several underlying layers—part-of-speech (POS) tags, named entities (NEs) with mappings to concept unique identifiers (CUIs), and two types of dependency parses (left). It then shows their translation into aligned layers (right).

Other than the base layer, each layer is an *artifact layer*, and is composed of a sequence of *artifacts*. For example, in Figure 2, *L*_*2*_ is named entities, and *L*_*2*_ = *l*_2,0_, *l*_2,1_, *l*_2,2_, … = C0032961, C0151526, C0011209, …. The artifacts are aligned with the base layer by storing 2 additional numbers: start index and length. Thus, a function *span* (·) on *l*_2,1_, a named entity spanning the words “preterm delivery,” would have *span*(*l*_2,1_) = (2,2) since the artifact concerns “preterm delivery” and starts at index 2 with a length of 2 tokens.

Additionally, we define a special *relation* artifact with a slight modification of other artifacts. For relation layers like *L*_3_ (stanford dependency parses, in Figure 2), artifacts are relations between two *other* artifacts’ locations. We will write these as *L*_3_ = *r*_3,0_, *r*_3,1_, *r*_3,2_, … to emphasize that these artifacts are relations. Then, *source* (·) and *target* (·) give the position and length of each of the relation’s arguments; *source*(*r*_3,0_) = (0,1) points from “pregnancy” to *target* (*r*_3,0_) = *base*(*r*_3,0_) = (3,1) “delivery.”

Note that the transformation to aligned language layers is slightly lossy. In Figure 2 the *L*_3_ artifacts corresponding to the head and dependent of each dependency relation are not preserved—only the *L*_0_-aligned spans. The loss is minimal in most NLP structures of interest.

### Scoring: Terms and phrases

In IR language models, it is common to rank according to score(*D,Q*) = *P*(*D*) *P*(*Q*|*D*). The conditional probability encapsulates the intuition that an ad hoc user trying to find document *D* will try to write an effective query *Q*. We focus our attention on the conditional probability *P*(*Q*|*D*).

This is most simply expressed in the standard *query likelihood* model with Dirichlet smoothing (with the parameter *μ*_D_), a baseline for language modeling approaches. We write single-**term queries** with the notation of the aligned layers above, where the *a* variable in *la* simply indicates that such term queries can be written for any single layer:

(1) P(la|D)t=#tD(la)+μ.#tD(la)|D|a|D|a+μ

where #*t*_D_ is the number of occurrences of the argument (a query term *l*_*a*_) in the document; #*t*_D_ is the number of occurrences of that argument in the whole collection. Similarly, |*D*|_*a*_ is the number of artifacts in the document from layer *l*_*a*_; |**D**|_α_ counts the same layer’s artifacts in the whole collection.

With a layered representation of both queries and documents, these term operators allow for querying of artifacts in any layer *L*_*a*_. The standard query likelihood model in other texts [5] is then just a special case of our term query utilizing only text layer artifacts *L*_0_ (i.e., from *L*_*text*_). The smoothed probability is also calculated with respect to frequencies in layer *L*_*a*_. Term queries do not require *aligned* layers — they consider each layer separately.

Probabilities for individual query terms can be calculated separately and combined to produce an overall probability. This is done implicitly, but can be made explicit with a **list query**; items in the list need not be in the same layer because they are each considered individually. A list query also groups the items in the list for use in other queries.

However, list queries ignore the collocation of layered artifacts; thus, we define the **phrase query.** A phrase is two or more artifacts from an arbitrary combination of layers, e.g., *l*_*ai*_*l*_*bj*_, the *i*^th^ artifact in *L*_*a*_ and the *j*^*th*^ artifact in *L*_*b*_. Phrases must be specified with ordering *o* as ordered or unordered (True or False), with a window size *w* to search within (measured according to positions in the base layer *L*_0_).

(2) $$P_{ph}\;\;\left(l_{ai}l_{bj}\ldots |D;o,w\right)=\frac{\#ph_{D}\left(l_{ai}l_{bj}\ldots \right)+\mu .\frac{\#ph_{D}\left(l_{ai}l_{bj}\ldots \right)}{{\left|D\right|}_{a}}}{\text{max}\left({\left|D\right|}_{a},{\left|D\right|}_{b},\ldots \right)+\mu }$$

The function #ph_*D*_ counts the number of cross-layer phrase matches in a document. Having aligned *l*_*ai*_*l*_*bj*_… to the base layer *L*_0_, we can check across layers for matches within a window. Window length is between the end of the first matched artifact, and the beginning of the last matched artifact (i.e., the last artifact only needs to have start index within the window length; it can end outside the window). Writing *l*_*ai*_*l*_*bj*_ illustrates that artifacts need not arise from the same layer, and will be in different positions within their respective layers (though aligned to the base layer *L*_0_). However, ordered phrases with *o*=True must additionally consist of only non-overlapping, sequential artifacts.

Whereas in single-layer queries, a probability estimate would consist of all items in that layer, there are now two or more layers to consider. It may at first seem that the denominator should be the product |*D*|_*a*_ *·* |*D*|_*b* ·_ …. This would mean: “out of all possible multi-layered phrases that could be constructed in document *D*, how likely is the specific construction we are looking for?” This choice of denominator would yield sparse probability estimates. Instead, we have chosen the size of the largest layer stipulated in the phrase. This answers the question: “of the artifacts in the largest layer, what proportion participate in a phrase consistent with the query?” This may be thought of as a backed-off estimate, and is more tractable. The choice of a denominator is an interesting area of further investigation beyond the scope of this work.

We should also note that this aligned-layer phrase queries subsume ordered and unordered phrases of feature-centric IR models [6], since those features are simply functions of *L*_0_.

### Implementation

The fundamental aims of the aligned-layer IR model require significant extension in any search engine. We implemented an aligned-layer language model via a plugin to Elasticsearch with multiple components, tested with Elasticsearch 1.7.2. We used cTAKES v3.2 as a preprocess [22], producing *character-aligned* artifacts (based on Apache UIMA^2^). Aligned-layer models are similar to searching over these UIMA-based data structures, but with scored ranking, across mutiple documents, and aligned on tokens instead of characters.

For indexing, each layer was represented as a field within a document. For our evaluation and analysis, we generated the an index with the following fields:

- *L*_*text*_: Word tokens, as identified by the cTAKES tokenizer; this is considered the base layer *L*0
- *L*_*cui*_: Concept Unique Identifiers (CUIs), as mapped from the UMLS Metathesaurus by cTAKES
- *L*_*tui*_: Type Unique Identifiers, a many-to-one mapping that groups concepts (CUIs) into semantic types (TUIs)
- *L*_*lemma*_: Normalized by a version of the National Library of Medicine’s (NLM) Lexical Variant Generator (LVG)
- *L*_*dep*_: Conll-U dependency parses from Clear Parser [23] trained on treebanked clinical text from Mayo Clinic
- *Lpos*: Part-of-speech tags produced by the OpenNLP^3^ MaxEnt POS tagger, trained on treebanked clinical text from Mayo Clinic

Each artifact was marked with its span (position and length, in tokens) via Lucene Payloads; this is a trivial marking for the base layer of text *L*_0_, but can be significant in other layers, such as for multi-word expressions in the CUI layer of Figures 1 and 2. In addition, the size of each field within a document was stored as metadata with that document at indexing time.

Queries are scored in Elasticsearch and Lucene via a highly optimized scoring interface. However, our scoring functions cannot be represented within that original structure; for example, the Lucene implementation of Dirichlet smoothing on a language model fails to divide by the collection frequency in some cases. Therefore, we implemented scoring via Elasticsearch Script Queries, which are typically used in a filtering context but here provide us the flexibility to score according to the models defined above.

Because they make use of aligned-layer terms and phrases, queries must be constructed and parsed differently than in other search systems. We implemented our own query parser in JavaCC to term and phrase operators in the aligned-layer query language.

### Task and Experiments

We provide a preliminary evaluation of aligned-layer language models, and of our Elasticsearch-based implementation in particular, on the task of patient cohort identification as exemplified in the 2011–2012 Text Retrieval Conference (TREC) Medical Records Track, or TREC-Med [18; 19]. In brief, there were 81 topics (34 from 2011, 47 from 2012) such as “patients with hearing loss,” and the task was to return lists of relevant patient visits (a de-identified surrogate for whole patient records) from among 17,198 possible visits. System-produced lists of visits were compared with human relevance judgments (which were gathered for the TREC-Med 2011–2012 competitions).

Table 1 shows how the same query is represented across our evaluated approaches. As a baseline test TXT, we used unstemmed, stopped TREC-Med queries with the standard query likelihood model -- namely, term queries on *L*_*text*_. A second baseline was CUI, where we used term queries on *L*_*cui*_.

In aligned-layer IR, term operators on multiple layers can be weighed together simultaneously for results. We replaced all named entity mentions in the query with the first-matched CUI, mixing the two layers in MIX. Since the cTAKES Named Entity Recognition (NER) module actually returned a set of CUIs per named entity, we included a model CUI-LS in which named entities were replaced with all of the CUIs associated with the same span, combined in a list. Next, we augmented the CUI list with a #ph containing *L*_*text*_ terms corresponding to any named entities, terming this PH-LS.

Searching for an optimal formulation and weighting of queries is outside the scope of this paper. However, for each of the above approaches, we also implemented a version with the Markov Random Field (MRF) “term” dependence model [24], which attempts to move beyond bag-of-words (BOW) models. Comparing the TXT-MRF row to the TXT row in Table 1 illustrates the behavior of this model: it tries out, with appropriate weights, the possibility that a multi-layered query may have phrases within it. We implemented a sequential dependence model with a limit of 5 aligned-layer components. These “term” dependence versions are marked as -MRF.

## Results

We used mean average precision (MAP) as the primary evaluation measure for evaluation. MAP provides a single-figure measure of quality across recall levels [25]. While TREC-Med 2011 reported bpref [26] as its primary evaluation metric, and TREC-Med 2012 reported infAP [27], we here report the mean average precision (MAP) due to its stability for both training and testing in previous work [28; 29]. Table 2 shows the MAP scores, where we have separated the topics from TREC-Med 2011 (left) and 2012 (right).

While this performance is below the state-of-the-art, it is interesting to note that it is layers with CUIs that obtain the highest performance in these tests (CUI-BOW on 2011 queries, and PH-LS-BOW on 2012 queries). Without text-layer query expansion, query logs, clickthrough data, or the like, another well-motivated, semantically-rich layer seems to be beneficial.

## Discussion

For aligned-layer language models, it is interesting to ask whether there is the possibility of fine-tuning results on individual queries. Here, then, we consider one of the worst-performing queries, topic 121, and seek to write aligned-layer queries that would improve those particular topics.

Topic 121 is “Patients with CAD who presented to the

Emergency Department with Acute Coronary Syndrome and were given Plavix.” The CUIs found were C0948089 (acute coronoary syndrome), C0010068 (coronary heart disease), and C0039082 (syndrome) – note the NER did not find the medication Plavix, or its generic form, clopidogrel. The highest scoring model on this topic was MIX-BOW=0.0639. In the event of a failed detection, we may consider using some kind of backoff model to less-specific expressions.

- In place of plavix, we searched for a dependency relationship where the head word has a lemma “give,” and the child is any word with a proper noun. It then weights that relation alongside the term plavix. This brought the MAP to 0.0803.
- In place of presented emergency department, we considered any verbs that might describe a patient’s arrival. We stipulated that there should be some verb in a dependency relationship with the phrase “emergency department.” This brought the MAP to 0.0867.

The multi-layered queries clearly allow for greater coarseness or fineness according to the needs of a query.

## Conclusion

We have introduced aligned-layer language models, a novel approach to IR that incorporates the output of other NLP systems. Core to this contribution are the definition of layers, alignment, and multi-layer scoring models. We have shown that this system can represent standard IR queries, as well as formulate multi-layered queries that were previously impossible. A case study demonstrates how the aligned-layer approach may feasibly be further extended to customize linguistic granularity to specific queries.

An open question of the proposed approach is how to design the layers for different corpora. Since different corpora contain variegated domain knowledge, we could design individualized layers for each corpus. In future work, we will further explore how our aligned-layer index can serve as a corpus analysis tool for quantitative representativeness of linguistic features; this would further drive a model to automatically design and select layers. In addition to this manual approach to exploring the linguistic content, we will learn parameters and weights for the model to optimize performance on IR tasks. Finally, we will release the code for aligned-layer language models to the open-source community

## Figures and Tables

**Table 1 -:** Eight versions of aligned-layer queries for Topic 125. #p are phrase queries, #l are list queries, others are term queries.

Layers	Query representation
TXT	coinfected hepatitis c hiv
CUI	cui:C0019196 cui:C0019158
MIX	coinfected cui:C0019158 hiv
PH-LS	coinfected #l(cui:C0019158| hepatitis) hiv
TXT-MRF	coinfected^0.85 hepatitiŝ0.85 ĉ0.85 hiv^0.85 #p(8|true|coinfected|hepatitis)^0.1 #p(8|false|coinfected|hepatitis)^0.05 #p(8|true|hepatitis|c)^0.1 #p(8|false|hepatitis|c)^0.05 #p(8|true|c|hiv)^0.1 #p(8|false|c|hiv)^0.05 #p(12|true|coinfected|hepatitis|c)^0.1 #p(12|falsejcoinfected| hepatitis|c)^0.05 #p(12|true|hepatitis|c|hiv)^0.1 #p(12|false|hepatitis|c|hiv)^0.05 #p(16|true|coin|fected|hepatitis|c|hiv)^0.1 #p(16|false|coinfected|hepatitis|c|hiv)^0.05
CUI-MRF	cui:C0019196^0.85 cui:C0019158^0.85 #p(8|true|cui:C0019196|cui:C0019158)^0.1#p(8|false|cui:C0019196|cui:C0019158)^0.05
MIX-MRF	coinfected^0.85 cui:C0019158^0.85 hiv^0.85 #p(8|true|coinfected|cui:C0019158)^0.1 #p(8|false|coinfected|cui:C0019158)^0.05 #p(8|true|cui:C0019158|hiv)^0.1 #p(8|false|cui:C0019158|hiv)^0.05 #p(12|true|coinfected|cui:C0019158|hiv)^0.1 #p(12|false|coinfected|cui:C0019158|hiv)^0.05
PH-LS-MRF	coinfected^0.85 #l(cui:C0019158|hepatitis)^0.85 hiv^8.85 #p (8|true|coinfected|#l(cui:C0019158|hepatitis))^0.1 #p(8|false|coinfected|#l(cui:C0019158|hepatitis))^0.05 #p(8|true|#l(cui:C0019158|hepatitis)|hiv)^0.1 #p(8|false|#l(cui:C0019158|hepatitis)|hiv)^0.05 #p(12|true|coinfected|#l(cui:C0019158|hepatitis)|hiv)^0.1

**Table 2 -:** Retrieval performance for a range of possible aligned-layer models

	2011		2012	
Model	BOW	MRF	BOW	MRF
TXT	0.2960	0.2936	0.2152	0.2224
CUI	0.3042	0.3119	**0.3126**	0.3101
MIX	0.2807	0.2847	0.2215	0.2167
CUI-LS	0.3076	0.3095	0.2161	0.2120
PH-LS	**0.3185**	0.3167	0.2172	0.2126

## Footnotes

1 http://ctakes.apache.org

2 http://uima.apache.org

3 https://opennlp.apache.org
